# Dose-volume analysis of planned versus accumulated dose as a predictor for late gastrointestinal toxicity in men receiving radiotherapy for high-risk prostate cancer

**DOI:** 10.1016/j.phro.2022.07.001

**Published:** 2022-07-16

**Authors:** Ashley L.K Ong, Kellie Knight, Vanessa Panettieri, Mathew Dimmock, Jeffrey K.L Tuan, Hong Qi Tan, Caroline Wright

**Affiliations:** a National Cancer Centre Singapore, Division of Radiation Oncology, Singapore; b Monash University, Department of Medical Imaging and Radiation Sciences, Clayton, Australia; c Alfred Hospital, Alfred Health Radiation Oncology, Melbourne, Australia; d Keele University, School of Allied Health Professions, Staffordshire, UK

**Keywords:** Accumulated dose, Predictive model, Gastrointestinal toxicity, High-risk prostate, Volumetric image-guidance

## Abstract

**Background and purpose:**

Significant dose deviations have been reported between planned (D_P_) and accumulated (D_A_) dose in prostate radiotherapy. This study aimed to develop multivariate analysis (MVA) models associating Grade 1 and 2 gastrointestinal (GI) toxicity with clinical and D_P_ or D_A_ dosimetric variables separately.

**Materials and methods:**

Dose volume (DV) metrics were compared between D_A_ and D_P_ for 150 high-risk prostate cancer patients. MV models were generated from significant clinical and dosimetric variables (p < 0.05) at univariate level. Dose-based-region of interest (DB-ROI) metrics were included. Model performance was measured, and additional subgroup analysis were performed.

**Results:**

Rectal D_A_ demonstrated a higher intermediate-high dose (V_30-65 Gy_ and DB-ROI at 15–50 mm) compared to D_P_. Conversely, at the very high dose region, rectal D_A_ (V_75 Gy_ and DB-ROI at 5–10 mm) were significantly lower. In MVA, rectal DB-ROI at 10 mm was predictive for Grade ≥ 1 GI toxicity for D_A_ and D_P_. Age, rectal D_A_ for D_0.03 cc_, and rectal D_P_ for DB-ROI 10 mm were predictors for Grade 2 GI toxicity. Subgroup analysis revealed that patients ≥ 72 years old and a rectal D_A_ of ≥ 78.2 Gy were highly predictive of Grade 2 GI toxicity.

**Conclusions:**

The dosimetric impact of a higher dose rectal dose in D_A_ due to volumetric changes was minimal and was not predictive of detrimental clinical toxicity apart from rectal D_0.03 cc_ ≥ 78.2 Gy for Grade 2 GI toxicity. The use of the DB-ROI method can provide equivalent predictive power as the DV method in toxicity prediction.

## Highlights

• Interfractional variations in organs at risk were observed in prostate radiotherapy.
• Rectal accumulated dose was significantly higher at the intermediate-high dose region.
• Rectal planned dose was significantly higher at the very high dose region.
• Dose>78.2 Gy to 0.03 cc of rectum was predictive of late Grade 2 toxicity.
• Patient age>72 years was predictive of late Grade 2 rectal toxicity.

## 1 Introduction

External beam radiotherapy combined with androgen deprivation therapy is the recommended clinical management for locally advanced high-risk prostate cancer (HR-PCa) [1]. As prostate cancer exhibits a low α/β value (1.5 Gy) which is comparable to that of late responding tissues [2], there’s a shift towards the use of dose escalation and hypofractionated regimens to improve the overall therapeutic ratio [3], [4]. This is especially critical for HR-PCa because of the high likelihood of mutation to a lethal phenotype that could reduce the biochemical and local control rate [5]. However, the drawback of these treatment schemes is often associated with a decline in patients’ quality of life as the incidence of reported gastrointestinal (GI) toxicity remains significant despite the use of advanced radiotherapy technologies [6], [7].

Significant volumetric variations of the organs at risk (OARs) that could affect the actual delivered dose during prostate RT have been reported [8], [9]. Small cohort studies demonstrated significant dose differences between accumulated dose (D_A_) obtained from either daily megavoltage computed tomography (MVCT) or repeated CT scans and planned dose (D_P_) [10], [11]. Majority of the large cohort studies focusing on the correlations between the rectal dose with the risk of late GI complications were mainly based on the dose distributions obtained with three-dimensional conformal radiation therapy (3D-CRT) techniques [12], [13]. Although some work has been done on evaluating the risk of GI toxicity with D_A_ generated based on patients’ cone-beam computed tomography (CBCT) scans, they were mainly performed on prostate only cases without the inclusion of pelvic lymph nodes (PLNs) [14], [15]. There is a paucity of work evaluating the dose difference in D_A_ and D_P_ on HR-PCa patients with PLNs irradiation using patients’ daily CBCT scans to assess the impact of inter-fractional organ motion on the risk of developing GI toxicity. In addition to the dosimetric variables, GI toxicity has also been demonstrated to be affected by patient-related factors, comorbidities, and intake of medications. Acute GI symptoms occurring within three months of treatment were also found to be significantly correlated with the incidence of late GI toxicity [7], [16].

In this study, we hypothesized that multivariate (MV) models generated that incorporate D_A_ together with clinical factors are more predictive than D_P_ values in associating late GI toxicity in HR-PCa with prophylactic pelvic lymph nodes (PLNs) irradiation. The objectives of this study were firstly, to evaluate the difference between D_A_ and D_P_ for the prostate and rectum using the previously developed dose accumulation workflow. Secondly, to construct MV models with Grade ≥ 1 and Grade ≥ 2 late GI toxicity as clinical endpoints whereby D_A_ and D_P_ were analysed separately with clinical variables. Lastly, subgroup analysis was performed on significant predictors that were correlated to the increased occurrence of late GI toxicity derived from the MV models.

## 2 Materials and methods

In this study, a total of 150 HR-PCa patients who were treated at our institution with PLN-irradiation between January 2016 and December 2019 were retrospectively recruited. The median follow-up (FU) for the entire cohort was 41.9 months, ranging from 16.3 to 62.1 months. Ethics approval was obtained from the centralised institutional review board (CIRB ref: 2019/2018). Patients’ characteristics, acute and late toxicity profiles are presented in Table 1.

**Table 1:** Patients’ characteristics, acute and late toxicity profiles.

Characteristics		N = 150 cases (%)
Age at diagnosis, yrs.; median [IQR]		72 [68–75]
BMI, kg/m^2^; median [IQR]		24.3 [22.5–26.4]
Gleason score; median [IQR]		8 [7–9]
≤ 7		62 (41.3 %)
> 7		86 (57.3 %)
Not known		2 (1.3 %)

cT-stage (AJCC 8th edition)		
≤ 2b		75 (50 %)
> 2b		75 (50 %)
Baseline PSA (ng/mL); median [IQR]		26.6 [11.7–55.5]

Medications		
Anti-hypertensive	Yes	74 (49.3 %)
	No	76 (50.7 %)
Metformin	Yes	33 (22 %)
	No	117 (78 %)
Statins	Yes	52 (34.7 %)
	No	98 (65.3 %)
TURP	Yes	13 (8.7 %)
	No	136 (90.7 %)
Not known		1 (0.7 %)
ADT	≤ 6 months	39 (26 %)
	> 6 months	111 (74 %)
RT prescription	≤ 74 Gy	88 (58.7 %)
	> 74 Gy	62 (41.3 %)

Organ volumes		
Prostate vol. (cm^3^); median [IQR]		31.9 [24.4–42.6]
Rectum vol. (cm^3^); median [IQR]		45.3 [36.2–57.4]

Overall acute toxicity		
	Grade ≥ 1	53 (35.3 %)
	Grade ≥ 2	12 (8 %)

Late toxicity		
Diarrhoea	Grade 1	1 (0.7 %)
	Grade 2	1(0.7 %)
Rectal hemorrhage	Grade 1	29 (19.3 %)
	Grade 2	9 (6 %)
Proctitis	Grade 1	16 (10.7 %)
	Grade 2	9 (6 %)
Overall late toxicity		
Grade ≥ 1		45 (30 %)
Grade ≥ 2		13 (8.7 %)

Abbreviations: BMI = body mass index, AJCC = American Joint Committee on Cancer antigen, PSA = prostate specific antigen; TURP = transurethral resection of the prostate; ADT = androgen deprivation therapy; GI = gastrointestinal; IQR = interquartile range.

### 2.1 CT-simulation and treatment planning

All patients were simulated in a supine position, using a leg immobiliser, with arms on their chests. Patients were advised by the radiation therapist on the bladder filling protocol (2–3 cups; 400–600 ml of water 30–60 mins before the procedure) and to empty their bowels before CT-simulation and during daily treatment. CT simulation was undertaken with 2.5 mm slice thickness (120kVp, GE LightSpeed RT 16).

A sequential (two-phase) treatment regimen was utilised, whereby a dose of 46–54 Gy in 23–27 fractions was prescribed to the prostate, seminal vesicles (SVs), and PLNs in Ph1. A coned-down Ph2 volume with a dose of 24–28 Gy in 12–14 fractions was delivered to the proximal 1 cm of the SVs and prostate, giving a total of 74–78 Gy. For Ph1, clinical target volumes (CTVs) were defined as the entire prostate and SVs with superior border set at L5/S1 interspace (including the distal common iliac, internal, and external iliac nodes). Ph1 planning target volume (PTV) was obtained by using an expansion margin of 5 mm posteriorly and 5–8 mm in all other directions. Ph2 PTV was generated by performing an isotropic 5 mm expansion from Ph2 CTV. All cases were planned with a 10 MV energy dual arc volumetric modulated arc therapy (VMAT) technique.

### 2.2 Dose based-region of interest (DB-ROI) structure

Apart from the standard DV variables, mean doses obtained from the alternative DB-ROI method were used as additional dosimetric variables for model building. The details of the generation of these structures were reported previously [17]. Briefly, DB-ROI structures were created by performing a volumetric expansion based on steps of 5 mm (5–50 mm) from the surface of the prostate gland. For each ROI expanded contour, the area of the previous smaller shape was subtracted from its area to yield strips of 5 mm structures at increasing distances from the prostate. The mean dose generated from the intersection of the 5 mm structures with the rectum was defined as D-x mm, whereby × ranged from 5 to 50 mm. This novel ROI method can account for the volumetric changes of the rectum at a specific distance from the prostate surface in a consistent manner, thus having the potential to overcome the uncertainties in visualizing the various rectal segments that were reported to contribute to toxicity [18], [19].

### 2.3 Treatment localisation and dose accumulation workflow

Daily CBCT scans were acquired in the treatment position using a half-fan mode (45 cm field-of-view, 120 kVp) scan, and reconstructed to 2.5 mm slice thickness (Varian on-board imaging v2.1, Varian Medical Systems, Palo Alto, CA) as part of the image localization protocol before treatment delivery. A total of 37–39 CBCTs images were available per patient to perform dose accumulation. A customised dose accumulation workflow was developed using MIM (MIMVista® v6.9, MIM Software Inc., Cleveland OH, USA) [20]. Details of the workflow construction and validation of an intensity-based deformable image registration (DIR) algorithm used in MIM have been described in our recent publication [17]. Within this workflow, fractional doses obtained from patients’ daily CBCT images were accumulated onto the pCT to create the final D_A_.

### 2.4 GI toxicity assessments and documentations

Late GI toxicity (diarrhoea, rectal haemorrhage and proctitis) was documented after three months post-RT, six-monthly for five years and yearly thereafter. In this study, the overall maximum occurrence of Grade ≥ 1 and Grade 2 GI toxicity determined at 2 years post-RT follow-up (FU) was considered as the clinical endpoints to be investigated. Majority of the toxicity records were documented electronically within the radiation-oncology-specific record and verification information system (Mosaiq; Elekta, Stockholm, Sweden). GI toxicity was graded according to the Radiation Therapy Oncology Group (RTOG) criteria. Approximately 20 % of the records were extracted and documented from free-text input from Mosaiq and patient’s physical case notes. Toxicity records were also re-populated, reviewed and verified in accordance with the National Cancer Institute Common Terminology Criteria for Adverse Events (version 4.03; CTCAE) by the radiation oncologist from the study team.

### 2.5 Statistical analysis

The primary clinical outcome of this study was the occurrence of Grade ≥ 1 and Grade 2 GI toxicity measured at two years post-RT FU. Descriptive statistics (e.g., means ± standard deviation, medians with interquartile ranges) were presented as appropriate. For the dosimetric analysis comparing D_P_ and D_A_ values, a parametric paired *t*-test was used after performing a normality test (Shapiro-Wilk test). A p-value < 0.05 being deemed significant. Highly correlated variables were filtered using Pearson correlation test (r ≥ 0.8) [21]. Univariate logistic analysis (UVA) was performed on individual clinical and dosimetric variables to define associations with late clinical endpoints. Significant variables at the UVA level (p < 0.05) were used for the subsequent multivariate logistic regression analysis (MVA) using an enter/remove method to identify the independent predictors for the final MV model, whereby p < 0.05 was considered statistically significant [22]. Results were reported as odds ratios, 95 % confidence intervals (CIs) and p-values.

Model performance was measured with respect to its calibration results and discriminative ability. Hosmer-Lemeshow p-value (p-HL) goodness of fit test was used to generate the calibration plot in which a p-value of > 0.05 indicates that the observed and predicted probability is similar, and therefore a good model fit is achieved [19]. For binary dependent variables, the observed outcomes were divided into quartiles to attain the observed probabilities and were plotted against the predicted probabilities. Model discrimination was assessed using the mean area under the receiver operating characteristic curve (AUC) to determine the overall model fit of the predictors with respect to the defined clinical endpoints. Achieving an AUC of ≥ 0.6 and having a minimum 95 % CI ≥ 0.5 were considered statistically significant by Gulliford et al [23]. Internal validation was accomplished using bootstrapping techniques to obtain the best fit predictors with associated 95 % CI as described in published studies [24], [25]. Lastly, subgroup analysis was conducted on MVA predictors using the AUC curve to determine the most relevant threshold differentiating the defined toxicity [26]. All analyses were performed using SPSS statistics (IBM Corp. v27.0. Armonk, NY) and R software (https://www.r-project.org/, version 4.0, Vienna, Austria).

## 3 Results

### 3.1 Dose-volume analysis between D_A_ and D_P_ for prostate and rectum

Absolute mean DV values for ${\overset{¯}{D}}_{A}$, ${\overset{¯}{D}}_{P}$ and the difference in mean dose; (${\overset{¯}{D}}_{A}$ - ${\overset{¯}{D}}_{P}$), Gy for the prostate and rectum are presented in Table 2. For the prostate, ${\overset{¯}{D}}_{D98\;\%\;}^{pros}$ coverage for D_A_ and D_P_ were statistically insignificant (p = 0.53). For the rectum, the D_A_ was significantly higher on average at the intermediate-high dose range; ${\overset{¯}{D}}_{A\;V30-65\;Gy}^{rect}$, with a>5 Gy difference being observed at the intermediate dose region (${\overset{¯}{D}}_{V35-45\;Gy}^{rect}$). On the contrary, at the very high dose region, D_P_ was significantly higher compared to D_A_ (${\overset{¯}{D}}_{D0.03\;Gy}^{rect}$, -<0.001 and ${\overset{¯}{D}}_{V75\;Gy}^{rect}$, p < 0.01).

**Table 2:** Evaluation of accumulated and planned prostate and rectum doses.

Organ	Parameters	${\overset{¯}{D}}_{P}$(±SD),Gy/%	${\overset{¯}{D}}_{A}$(±SD),Gy/%	${\overset{¯}{D}}_{A}$- ${\overset{¯}{D}}_{P}$(±SD), Gy/%	95 % CI of the diff.	p - value
Prostate	D98 % [Gy]	76.5 (2.1)	76.6 (2.3)	0.1 (1.0)	0.12–0.21	p = 0.53
Rectum	Dmean [Gy]	46.0 (4.6)	47.7 (4.8)	1.7 (2.3)	1.36–2.10	p < 0.001
	D0.03 cc [Gy]	77.4 (2.3)	76.5 (2.5)	−0.8 (1.2)	0.66–1.04	p < 0.001
	V30 Gy [%]	81.8 (12.2)	85.8 (10.5)	4.2 (4.5)	3.30–4.74	p < 0.001
	V35 Gy [%]	70.1 (14.2)	75.6 (13.1)	5.5 (5.1)	4.71–6.33	p < 0.001
	V40 Gy [%]	57.8 (13.7)	63.9 (13.5)	6.1 (5.3)	5.28–7.00	p < 0.001
	V45 Gy [%]	46.5 (12.3)	52.3 (13.0)	5.8 (5.5)	4.86–6.64	p < 0.001
	V50 Gy [%]	37.2 (11.0)	41.2 (12.3)	4.8 (5.8)	3.87–5.74	p < 0.001
	V55 Gy [%]	29.9 (9.5)	33.6 (11.2)	3.8 (5.8)	2.84–4.72	p < 0.001
	V60 Gy [%]	23.6 (8.1)	26.2 (10.0)	2.6 (5.7)	1.68–3.50	p < 0.001
	V65 Gy [%]	17.9 (6.8)	19.1 (8.7)	1.2 (5.3)	0.34–2.06	p < 0.01
	V70 Gy [%]	12.4 (5.4)	12.0 (7.2)	−0.4 (4.7)	0.39–1.12	p = 0.35
	V75 Gy [%]	4.8 (4.4)	3.8 (4.7)	−1.0 (3.0)	0.50–1.46	p < 0.001

Abbreviations: ${\overset{¯}{D}}_{A}$= mean accumulated dose, ${\overset{¯}{D}}_{P}$= mean planned dose; SD = standard deviation; Dmean = mean dose; Dx [Gy] = dose [Gy] received by the specified × volume (%); Vx [%] = volume of the organ [%] receiving the specified × dose (Gy); CI = confidence interval.

### 3.2 Dose-based ROI analysis between D_A_ and D_P_ for the rectum

The absolute mean DV values for rectal ${\overset{¯}{D}}_{A}$, ${\overset{¯}{D}}_{P}$ and the difference in dose; (${\overset{¯}{D}}_{A}$ - ${\overset{¯}{D}}_{P}$), Gy were calculated for all patients per ROI; ${\overset{¯}{D}}_{ROI\;5-50\;mm}^{rect}$ (5–50 mm from the prostate surface) and were shown in Table 3. ${\overset{¯}{D}}_{A\;ROI\;15-50mm}^{rect}$ received a higher dose (1.4 – 3.6 Gy) compared to ${\overset{¯}{D}}_{P\;ROI\;15-50mm}^{rect}$ and were statistically significant (p < 0.001). On the contrary, at ${\overset{¯}{D}}_{ROI\;5-10\;mm}^{rect}$, D_A_ on average was significantly lower compared to D_P_ (p < 0.001)_._

**Table 3:** Evaluation of accumulated and planned dose delivered to rectal ROIs.

Rectum (mm)	${\overset{¯}{D}}_{P}$(±SD), Gy	${\overset{¯}{D}}_{A}$(±SD), Gy	${\overset{¯}{D}}_{A}$- ${\overset{¯}{D}}_{P}$ (±SD), Gy	95 % CI of the difference	p-value
5	75.2 (9.0)	73.8 (9.1)	−1.4 (1.9)	1.10–1.73	p < 0.001
10	70.6 (2.9)	69.3 (4.2)	−1.3 (3.4)	0.80–1.89	p < 0.001
15	57.4 (4.7)	58.9 (5.8)	1.4 (4.3)	0.74–2.13	p < 0.001
20	46.3 (5.6)	48.5 (6.7)	2.2 (3.9)	1.55–2.81	p < 0.001
25	39.4 (6.2)	41.7 (7.0)	2.3 (3.4)	1.75–2.85	p < 0.001
30	34.7 (6.6)	37.6 (7.4)	2.9 (3.6)	2.27–3.44	p < 0.001
35	31.4 (7.1)	35.0 (8.1)	3.6 (4.0)	2.90–4.19	p < 0.001
40	28.6 (8.1)	32.3 (9.5)	3.7 (4.8)	2.94–4.48	p < 0.001
45	26.2 (10.5)	29.5 (11.9)	3.3 (6.0)	2.32–4.25	p < 0.001
50	22.0 (13.8)	24.4 (15.2)	2.4 (6.9)	1.27–3.48	p < 0.001

Abbreviations: ROI = region of interest; ${\overset{¯}{D}}_{A}$ = mean accumulated dose, ${\overset{¯}{D}}_{P}$= mean planned dose; SD = standard deviation; CI = confidence interval.

### 3.3 MV modelling and model performance evaluation.

**Fig. 1:**
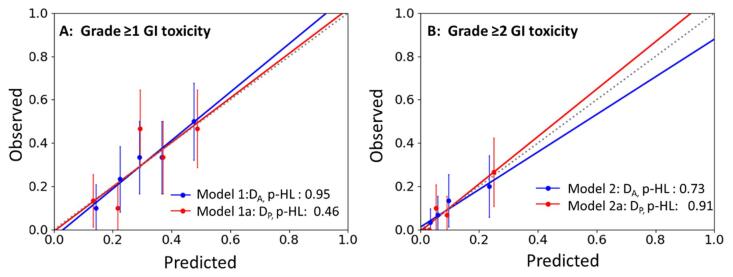
Calibration plots (predicted vs observed probabilities) for Grade ≥ 1 GI (A) and Grade ≥ 2 GI (B). The 45° dotted line represents the reference line where y = x.

In the MV modelling, the correlations of clinical variables were tested separately with each of the two dosimetric variables D_A_ and D_P_ (see Table 4). Four statistically significant models (p < 0.05) were generated based on the significant predictors obtained from the UVAs, associated with the development of Grade ≥ 1 and Grade 2 late GI toxicity. A complete list of UVA results is available in supplemental table (Table S1). All statistically significant MV models achieved an AUC of ≥ 0.6 and a minimum 95 % CI ≥ 0.5, meeting the stated criteria for good model performance (Table S2). Similarly, all four models attained a p-HL of ≥ 0.5 as demonstrated in the calibration plots (Fig. 1.), suggesting that the predicted probability coincides well with the observed events.

**Table 4:** Final MV models using statistically significant predictors (p < 0.05) from UVA.

MV Models	Clinical and D_A_ or D_P_	Variables	OR	95 % CI	p-value
Model 1	D_A_, Grade ≥ 1	${\overset{¯}{D}}_{A\;ROI\;10\;mm\;(Gy)}^{rect}$	1.13	1.03–1.24	p < 0.01
		${\overset{¯}{D}}_{A\;V35\;Gy\;(\%)\;}^{rect}$	0.97	0.94–0.99	p < 0.05

Model 1a	D_P_, Grade ≥ 1	${\overset{¯}{D}}_{P\;ROI\;10\;mm\;(Gy)}^{rect}$	1.17	1.03–1.34	p < 0.05
		${\overset{¯}{D}}_{P\;V35\;Gy\;(\%)\;}^{rect}$	0.96	0.94–0.99	p < 0.01

Model 2	D_A_, Grade ≥ 2	Age, yrs.	1.15	1.04–1.29	p < 0.01
		${\overset{¯}{D}}_{A\;D0.03\;cc\;(Gy)}^{rect}$	1.34	1.02–1.77	p < 0.05

Model 2a	D_P_, Grade ≥ 2	Age, yrs.	1.15	1.04–1.29	p < 0.01
		${\overset{¯}{D}}_{P\;ROI\;10\;mm\;(Gy)}^{rect}$	1.35	1.06–1.71	p < 0.01

Abbreviations: D_A_ = accumulated dose; D_P_ = planned dose; GI = gastrointestinal; ROI = region of interest; OR = odds ratio; ${\overset{¯}{D}}_{A/P\;ROI\;x\;mm\;(Gy)}^{rect}$ = mean rectal dose for D*_A_* or D*_P_* at ROI x mm distance.${\overset{¯}{D}}_{A/P\;Vx\;Gy\;(\%)\;}^{rect}$ = mean rectal volume (%) for D*_A_* or D*_P_* receiving the specific × dose (Gy); ${\overset{¯}{D}}_{A/P\;Dx\;cc\;(Gy)}^{rect}$= mean rectal dose (Gy) received by D_A_ or D_P_ for the specified × volume (cc).

### 3.4 MV regression analysis of Grade 1 and Grade 2 GI toxicity at 2 years post-RT FU

For Grade ≥ 1 late GI toxicity using clinical and D_A_ or D_P_ assessment, ${\overset{¯}{D}}_{ROI\;10\;mm}^{rect}$ (D_A_; OR: 1.13 vs D_P_; OR: 1.17) and ${\overset{¯}{D}}_{V35\;Gy}^{rect}$ (D_A_; OR: 0.97 vs D_P_; OR: 0.96) were significant dosimetric predictors associated with the development Grade ≥ 1 GI toxicity for D_A_ and D_P_. The same AUC value of 0.67 was achieved for Models 1 and 1a. None of the clinical predictors were significant for these models. For Grade ≥ 2 late GI toxicity using clinical and D_A_ and D_P_ assessment, age was a significant clinical predictor (OR: 1.15, p < 0.01) in Models 2 and 2a. Additionally, ${\overset{¯}{D}}_{A\;D0.03\;cc}^{rect}$ (Model 2, D_A_; OR: 1.34) and ${\overset{¯}{D}}_{P\;ROI\;10\;mm}^{rect}$ (Model 2a, D_P_; OR: 1.35) remained as significant dosimetric predictors with age in both models; attaining overall high AUC values of 0.78 and 0.81 for Model 2 and 2a respectively (Table 4).

### 3.5 Subgroup analysis

Based on the MV models, five predictors defined by an associated increased in occurrence of late GI toxicity were further evaluated to determine the optimal cut-off values using the AUC curve. Cut-off values with the highest sensitivity and lowest specificity were selected. As shown in Table S3, patients who were ≥ 72 years old (AUC: 0.86, p < 0.05; 95 % CI: 0.53–0.82) were 3.6 times more likely to experience Grade ≥ 2 GI toxicity at 2 years post-RT FU (UVA: p < 0.05, CI: 0.95–13.6) compared to those younger than 72 years old. Similarly, patients with an accumulated dose, D_A_ ≥ 78.2 Gy (AUC: 0.67, p < 0.05; 95 % CI: 0.55–0.80) delivered to 0.03 cc of the rectum were 4.8 times more likely to suffer from Grade ≥ 2 GI toxicity (UVA: p < 0.01, CI: 1.39–16.29) compared to those receiving<78.2 Gy to 0.03 cc of the rectal volume. The rest of the predictors remained statistically insignificant in UVA after applying the selected cut-off values for the defined toxicity.

## 4 Discussion

This study hypothesised that for HR-PCa patients undergoing PLNs irradiation, MV models obtained using a dose accumulation workflow to generate D_A,_ together with clinical factors, are more predictive than D_P_ for late GI toxicity. Apart from incorporating the pertinent clinical variables to predict the occurrence of Grade ≥ 1 and Grade 2 late GI toxicity, D_A_ received by the rectum (which accounted for daily inter-fractional organ motion) further enhanced the predictive power of the developed models [15]. This study is one of the largest dose comparative series to date, incorporating D_A_ as the dosimetric variable used to perform MV modelling for late GI toxicity in HR-PCa patients treated with the highly conformal VMAT technique.

For Models 1 and 1a, the ${\overset{¯}{D}}_{ROI\;10\;mm}^{rect}$ parameter represents the segment of the rectum that is close to the prostate high dose region and was associated with an increased risk of Grade ≥ 1 GI toxicity [27]. ${\overset{¯}{D}}_{V35Gy}^{rect}$ for D_A_ and D_P_ was significant in Grade ≥ 1 GI toxicity in MV modelling. Splashes of low doses to the rectum, which increases due to the inclusion of PLNs irradiation has negligible impact on the risk of having Grade ≥ 1 GI toxicity as demonstrated in this study. Despite having a significantly higher percentage of rectal volume receiving 35 Gy in D_A_, which could be attributed to the occurrence of rectal distension at the superior portion of the rectum as previously reported [17], [28], it does not have a parallel impact on rectal toxicity. Similarly, results reported by Vargas et al [29], on associating DV metrics with late Grade 2 GI toxicity concluded that relative volume of rectum irradiated to ≤ 40 Gy was not predictive for this toxicity endpoint.

For Models 2 and 2a, advanced age is highly associated with the increasing risk of Grade 2 GI toxicity, which is in parallel to reported studies [30], [31]. In Model 2, the interactions with age and ${\overset{¯}{D}}_{A\;D0.03\;cc}^{rect}$ demonstrated good model performance (AUC, 0.78, p < 0.001). Age and high dose to small volume of the rectum were often being reported as having a strong correlation to late Grade 2 GI toxicity [32]. Even though ${\overset{¯}{D}}_{A\;D0.03\;cc}^{rect}$ was significantly lower compared to D_P_ in DV analysis due to potential reduction in rectal volume along the segment that lies directly posterior to the prostate, it has remained as a strong predictor in MV analysis. This further confirmed the underlining serial-like behaviour of the rectum that is highly correlated to the small rectal volume receiving ≥ 74 Gy [28]. In Model 2a, age and ${\overset{¯}{D}}_{P\;ROI\;10\;mm}^{rect}$ were highly predictive of patients suffering from late Grade 2 toxicity, achieving similar good model performance as Model 2 (AUC, 0.81, p < 0.001). The use of alternative DB-ROI structures that are closer to the prostate (5–10 mm) might be equivalent to the very high dose range (V70-75 Gy) in routine DV scenarios. Educating the importance of having an empty rectum, dietary interventions and the use of laxatives have been suggested to maintain the consistency of a small rectal volume [33], thus minimising the risk of late GI toxicity.

In the subgroup analysis, ${\overset{¯}{D}}_{A\;D0.03\;cc}^{rect}$ was the only significant predictor for Grade ≥ 2 late GI toxicity whereby a dose limit of 78.2 Gy was recommended with a 4.8 times greater probability of developing this clinical endpoint for every 1 Gy increase in dose. This dose limit is parallel to the recommended values reported in studies investigating DV limits with GI toxicity [6], [28]. Interfractional rectal motion might be minimal beyond the dose limit of 78.2 Gy and thus the toxicity event rates were low. Age ≥ 72 years (p < 0.05) was the only significant clinical predictor for Grade ≥ 2 late GI toxicity. Results obtained from a similar study conducted by Pederson et al. [34] also found that advanced age (≥70 years) correlates with an increased risk of late GI toxicity. Often, age is among the most important prognostic factors in guiding decision making with regards to patient expectations in terms of treatment aggressiveness with the aim for cure and expected treatment-related toxicity/ quality of life.[35].

This study has some limitations. Firstly, external validation was not performed on the developed models. However, internal validation was conducted to examine model performance and met the robustness criteria whereby an average AUC of ≥ 0.6 were achieved in all the MV models.

Secondly, the relatively short late toxicity timeframe at two years post-RT FU might not be sufficient to capture the late toxicity effects as a latency period of delayed occurrence have been reported to be greater than 5 years [3], [36], [37]. However, in this study, as all patients have completed their 2 years post-RT FU, the derived models are highly predictive in determining late GI toxicity in HR-PCa patients at a two-year time-point.

The novelty of this work was to use the mean dose derived from the DB-ROIs as surrogates for various dose spectrums. These parameters were found to be highly predictive of late GI toxicity compared to the standard DV metrics as observed in the final MV models (Table 5). DB-ROI structures can be generated automatically in an unbiased manner using the prostate gland as the base structure and accounting for the patient-specific volumetric organ variations during RT. DB-ROI structures can complement the standard values for toxicity association, thereby reducing the challenges in identifying the specific region of the rectum that is associated with the defined toxicity [19].

Despite the higher dose at the intermediate-high dose region obtained in D_A_, it does not translate into a corresponding increase in late Grade 2 GI toxicity. The use of modern radiotherapy techniques such as the use of more accurate magnetic resonance imaging-based target definition and the ability to generate smaller PTV margins from the utilisation of image guidance might have rendered the impact of volumetric changes of the OARs during RT insignificant. A larger sample size with a longer follow up will be necessary to determine the impact of volumetric changes on associated late toxicity and to substantiate the result of ${\overset{¯}{D}}_{A\;D0.03\;cc}^{rect}$ being highly predictive of late Grade 2 GI toxicity. Lastly, this study could be further expanded to incorporate the use of dose surface maps for D_A_ and D_P_ and correlate with toxicity as voxel-level dose have been reported to improve the accuracy in identifying heterogeneous areas of heightened dose sensitivity [15], [38].

In conclusion, the developed MV models for HR-PCa treated using inverse planning techniques to predict the risk of late GI toxicity at two years post-RT FU will be able to provide guidelines to facilitate dose escalation. In particular, this work has shown that patient age >72 years and has an accumulated dose of > 78.2 Gy received by 0.03 cc of the rectum are significant predictors of Grade ≥ 2 late GI toxicity.

## Funding statement

This work was partially funded by Monash University, Medical Imaging and Radiation Sciences Higher Degree Research student fund, 2021 and the Duke-NUS Oncology Academic Program Goh Foundation Proton Research Programme (08/FY2021/EX(SL)/92-A146).

## Declaration of Competing Interest

The authors declare that they have no known competing financial interests or personal relationships that could have appeared to influence the work reported in this paper.
