# Supplementary material for: Dose-volume analysis of planned versus accumulated dose as a predictor for late gastrointestinal toxicity in men receiving radiotherapy for high-risk prostate cancer
Source: Phys Imaging Radiat Oncol. 2022 Jul 16;23:97–102. doi: 10.1016/j.phro.2022.07.001 (PMC9307677; doi:10.1016/j.phro.2022.07.001)
Supplement: Supplementary data 1 [file mmc1.pdf]

Supplemental Material

Dose-volume analysis of planned versus accumulated dose as a predictor for late gastrointestinal toxicity in men receiving radiotherapy for high-risk prostate cancer

Supplemental table S1. Univariate binary logistic regression was used to analyse individual predictors with the defined late GI toxicity endpoints. A p-value of <0.05 was considered statistically significant.

**Univariate analysis**	**Grade ≥1 late GI toxicity**			**Grade 2 late GI toxicity**		
	Grade <1 (N=105); Grade ≥1 (N=45)			Grade <2 (N=137); Grade ≥2 (N=13)		
**Clinical covariates**	**OR**	**95% CI**	**p-value**	**OR**	**95% CI**	**p-value**
Age, yrs.				1.15	1.04 - 1.28	0.01
**Dosimetric covariates for D_A**						
Prostate $D_{98\%}$ (Gy)				1.35	1.02 - 1.77	0.03
**Rectum**						
$D_{0.03\ cc}$ (Gy)				1.32	1.02 - 1.72	0.04
$V_{35\ Gy}$ (%)	0.97	0.95 – 0.99	0.049			
**DB-ROI (Gy)**						
D- 5 mm				1.31	1.03 - 1.68	0.03
D- 10 mm	1.11	1.01 - 1.21	0.03			
**Dosimetric covariates for D_P**						
**Rectum**						
$V_{35\ Gy}$ (%)	0.97	0.95 – 0.99	0.02			
**DB-ROI (Gy)**						
D- 5 mm				1.38	1.03 - 1.86	0.03
D- 10 mm	1.15	1.01 - 1.31	0.04	1.35	1.06 - 1.70	0.01

Abbreviations: OR = odds ratio, CI = confidence interval, D_P = Planned dose, D_A = Accumulated dose, BMI = body mass index, GS = Gleason score, BL PSA = baseline prostate specific antigen, TURP = transurethral resection of the prostate, ADT = androgen deprivation therapy, DB-ROI = dose-based region of interest

Supplemental table S2. Mean area under the ROC curve (AUC) obtained for each model to evaluate model performance of the MV models.

MV Models	**Model performance**							
	Clinical vs D_A or D_P	p-HL	R^2	AUC	p-value	95% CI	Sensitivity	1-Specificity
Model 1	D_A, Grade ≥1	0.95	0.10	0.67	p<0.001	0.58 - 0.77	0.71	0.42
Model 1a	D_P, Grade ≥1	0.46	0.11	0.67	p<0.001	0.58 - 0.76	0.78	0.40
Model 2	D_A, Grade ≥2	0.73	0.18	0.78	p<0.001	0.66 - 0.90	0.92	0.42
Model 2a	D_P, Grade ≥2	0.91	0.21	0.81	p<0.001	0.72 - 0.91	0.92	0.39

Abbreviations: MV = multivariate, D_A = accumulated dose, D_P = planned dose, GI = gastrointestinal; p-HL = Hosmer-Lemeshow p-values for goodness of fit test; AUC = area under the receiver operator curve; R^2 = Pseudo R^2.

Supplemental table S3. Subgroup analysis using the mean area under the ROC curve (AUC) to determine the optimal cut-off values and application of these threshold values on UVA. Statistically significant p-values were in bold.

		**AUCs for determination of optimal cut-off values**						**Cut-off value on UVA**		
Models	Parameters	AUC	95% CI	p-value	Cut-0ff value	Sensitivity	1-Specificity	OR	95% CI	p-value
Model 1	$\overline{\mathrm{D}}_{A\ \mathrm{ROI\ 10\ mm\ (Gy)}}^{\mathrm{rect}}$	0.61	0.51-0.71	p≤0.05	69.8 Gy	0.60	0.44	1.92	0.95-3.91	p=0.07
Model 1a	$\overline{\mathrm{D}}_{P\ \mathrm{ROI\ 10\ mm\ (Gy)}}^{\mathrm{rect}}$	0.59	0.48-0.69	P=0.10	70.9 Gy	0.53	0.46	1.47	0.73-2.96	P=0.29
Model 2/2a	Age	0.68	0.53-0.82	p≤0.05	72 yo	0.77	0.48	3.59	0.95-13.60	**P<0.05**
Model 2	$\overline{\mathrm{D}}_{A\ \mathrm{D0.03\ cc\ (Gy)}}^{\mathrm{rect}}$	0.67	0.55-0.80	p≤0.05	78.2 Gy	0.69	0.32	4.76	1.39-16.29	**P<0.01**
Model 2a	$\overline{\mathrm{D}}_{P\ \mathrm{ROI\ 10\ mm\ (Gy)}}^{\mathrm{rect}}$	0.71	0.56-0.86	p≤0.01	71 Gy	0.77	0.43	2.12	0.66-6.80	P=0.21

Abbreviations: ROI = region of interest, ROC= receiver operating characteristic curve, AUC = mean area under the receiver operator curve, UVA = univariate analysis, $\overline{\mathrm{D}}_{A/P\ \mathrm{ROI\ x\ mm\ (Gy)}}^{\mathrm{rect}}$ = mean rectal dose for D_A or D_P at ROI x mm distance, $\overline{\mathrm{D}}_{A/P\ \mathrm{Dx\ cc\ (Gy)}}^{\mathrm{rect}}$= mean rectal dose (Gy) received by D_A or D_P for the specified x volume (cc).
